# Neurodegeneration in Multiple Sclerosis: The Role of Nrf2-Dependent Pathways

**DOI:** 10.3390/antiox11061146

**Published:** 2022-06-10

**Authors:** Paloma P. Maldonado, Coram Guevara, Margrethe A. Olesen, Juan Andres Orellana, Rodrigo A. Quintanilla, Fernando C. Ortiz

**Affiliations:** 1 Mechanisms of Myelin Formation and Repair Laboratory, Instituto de Ciencias Biomédicas, Facultad de Ciencias de Salud, Universidad Autónoma de Chile, Santiago 8910060, Chile; palomix.mr@gmail.com (P.P.M.); coram.guevara@cloud.uautonoma.cl (C.G.); 2 Laboratory of Neurodegenerative Diseases, Instituto de Ciencias Biomédicas, Facultad de Ciencias de la Salud, Universidad Autónoma de Chile, Santiago 8910060, Chile; margrethe.olesen@cloud.uautonoma.cl (M.A.O.); rodrigo.quintanilla@uautonoma.cl (R.A.Q.); 3 Departamento de Neurología, Escuela de Medicina y Centro Interdisciplinario de Neurociencias, Facultad de Medicina, Pontificia Universidad Católica de Chile, Santiago 8330024, Chile; jaorella@uc.cl

**Keywords:** multiple sclerosis, Nrf2-dependent pathways, neuroinflammation, pannexin-1, glial cells, reactive oxygen species, mitochondria

## Abstract

Multiple sclerosis (MS) encompasses a chronic, irreversible, and predominantly immune-mediated disease of the central nervous system that leads to axonal degeneration, neuronal death, and several neurological symptoms. Although various immune therapies have reduced relapse rates and the severity of symptoms in relapsing-remitting MS, there is still no cure for this devastating disease. In this brief review, we discuss the role of mitochondria dysfunction in the progression of MS, focused on the possible role of Nrf2 signaling in orchestrating the impairment of critical cellular and molecular aspects such as reactive oxygen species (ROS) management, under neuroinflammation and neurodegeneration in MS. In this scenario, we propose a new potential downstream signaling of Nrf2 pathway, namely the opening of hemichannels and pannexons. These large-pore channels are known to modulate glial/neuronal function and ROS production as they are permeable to extracellular Ca^2+^ and release potentially harmful transmitters to the synaptic cleft. In this way, the Nrf2 dysfunction impairs not only the bioenergetics and metabolic properties of glial cells but also the proper antioxidant defense and energy supply that they provide to neurons.

## 1. Introduction

Multiple sclerosis (MS) is an irreversible, progressive pathology that originates in the autoimmune attack of T and B lymphocytes against self-antigens of the myelin and oligodendrocytes (OLs). This phenomenon leads to axonal degeneration, neuronal death, and several neurological symptoms, including mobility and sensory impairment, fatigue, and temporary vision loss. MS encompasses demyelinated insults at early stages, followed by a spontaneous, yet incomplete or poor myelin repair process at the lesion site (i.e., remyelination) [1]. Extensive attention has been given to understanding the cellular mechanisms underlying the demyelination and myelin repair processes. However, although axonal degeneration relies on demyelination and poor trophic support from OLs [2,3], axonal and neuronal injury can also occur without demyelination [2]. This shows that other processes that go hand in hand with demyelination could account for the neurodegenerative features that correlate with the MS clinical hallmarks. The cornerstones of remyelination are glial cell-mediated neuroinflammation, the over-production of mitochondrial reactive oxygen species (ROS), and mitochondrial dysfunction. Importantly, these features occur in other neurodegenerative disorders such as Alzheimer’s disease (AD), Parkinson’s disease (PD), and amyotrophic lateral sclerosis (ALS) [4]. Another aspect common to neurodegenerative and/or neurological disorders, including MS, is the deficiency of the Nrf2 (nuclear factor erythroid 2-related factor 2) signaling pathway, a critical antioxidant transcription factor that prevents mitochondrial failure, oxidative stress, and neuroinflammation.

In this brief review, we compile and discuss the evidence supporting a pivotal role of disrupted Nrf2 signaling in orchestrating glia-mediated ROS increase and mitochondrial dysfunction at the onset of MS.

## 2. Multiple Sclerosis

Myelin is a specialized membrane that enwraps axons, making fast saltatory action potential propagation possible and providing metabolic support to the neurons [1,5]. A lack of myelin (i.e., demyelination) across the central nervous system (CNS) white matter tracts (i.e., corpus callosum, optic nerve, cerebellar white matter, and spinal cord) characterizes MS. Demyelination leads, in turn, to axonal degeneration, neuronal death, and several neurological disabilities that manifest in very variable symptoms [1,6,7]. This neurodegenerative disease affects around 2.5 million people worldwide [6], representing the second most common cause of disabilities in the young adult population. The relapsing–remitting form of MS (RRMS) is the most common MS type present in the population, accounting for around 85% of the cases. RRMS is characterized by the appearance of attacks—or relapses—where patient symptoms worsen, followed by periods of remission of these neurological manifestations [6]. Over 10 to 15 years of evolution, about 40–50% of patients have developed the secondary progressive form of the disease (SPMS), accumulating disability without remission. Only about 15% of patients exhibit the primary progressive (PPMS) form of the disease at the onset, featured by the progressive accumulation of neurological damage. Thus, there are three major clinical forms of MS: RRMS, SPMS, and PPMS [1,6,8].

The early years of MS encompass demyelinated lesions followed by spontaneous, although incomplete, myelin repair processes. In these lesions, OPCs differentiate into remyelinating oligodendrocytes, which are the myelin-forming cells of the CNS. However, this process usually results in incomplete myelinization or the production of low-quality myelin. This failure in remyelination precedes a progressive axon degeneration aggravating the neurological symptoms in MS patients, such as mobility and sensory impairment, fatigue, and temporary loss of vision, among many others leading to progressive disease [1,5,6,9,10,11]. Thus, primary demyelination leads to neurodegeneration, causing devastating neurological damage and disability in MS patients. Unfortunately, the mechanisms of neurodegeneration behind MS progression are only partially understood and treated. We speculate that, similar to other neuroinflammatory-characterized diseases, the microenvironment generated during the demyelination process could be a critical context explaining neurodegenerative features.

In these days, the successive increase in neurodegenerative features is clinically estimated by indirect measurements of relevant parameters, such as disability accumulation (expanded disability status scale—EDSS), cognitive tests, and brain atrophy index based on magnetic resonance imaging (MRI) [12]. Nevertheless, reliable biomarkers indicating an accurate estimation of quality and degree of neurodegeneration during MS progression are still missing [13,14].

## 3. Glial Cell-Mediated Neuroinflammation

Neuroinflammation is the complex innate immune response (sometimes adaptive) against internal or external agents, aiming to resist or resolve harmful threats to restore homeostasis [4,15,16]. Two glial cells, microglia and astrocytes, are cornerstones in this process as they restrain infection and eliminate pathogens, cell debris, and misfolded proteins. During intense pathological conditions, neuroinflammation becomes persistent and detrimental to proper brain function, with the above glial cells being the architects of this phenomenon. In particular, they experience a long-lasting morphological, molecular, and functional change called “reactive gliosis”. While this process is an adaptive mechanism necessary for limiting acute injury and favoring wound repair, when it persists, it can become a detrimental response if these glial cells neglect their supportive role toward neurons. In MS, the first autoimmune attack against myelin and OLs settles a chronic and escalating neuroinflammatory niche that fosters the activation of microglia, astrocytes, and perivascular macrophages [1]. At one end, the dysfunctional activation of microglia contributes to MS disease pathology by promoting the release of proinflammatory cytokines, chemokines, ROS, and glutamate, a subject that has been extensively studied [16,17,18,19,20] for a recent review see [16].

On the other hand, astrocytes are activated either directly by the initial pathogenic immune insult or indirectly by microglial mediators such as IL-1β [21] and interferon-γ (IFN-γ) [22]. In that scenario, astrocytes release IL-23, inducing CD4^+^T differentiation and maintaining the proinflammatory Th17 lineage [23], an essential immune cell type in CNS demyelinating diseases [24]. Most of these inflammatory cascades enhance ROS production by affecting mitochondrial bioenergetics and the antioxidant response of neurons. Accordingly, oxidative stress and mitochondrial failure markers are present in post mortem brain tissue, cerebrospinal fluid (CSF), and serum samples from MS patients and animal models [13,25].

Although significant efforts have been made to understand and ameliorate the acute neuroinflammatory components of MS, the pathophysiological mechanisms of inflammation, mitochondrial dysfunction, and oxidative stress as well as the concomitant neurodegeneration are still crucial parts of a puzzle that have not yet been completely defined.

## 4. Mitochondria and Neuronal Function

Mitochondria are pivotal organelles in charge of energy supply by controlling adenosine triphosphate (ATP) production, redox balance, and the intracellular free Ca^2+^ concentration ([Ca^2+^]_i_) in neuronal cells [26]. These processes are critical to sustaining proper brain cell communication at the synaptic cleft [26,27] The structure of the mitochondria consists in an outer membrane (OMM) and an inter membrane (IMM) that allow the passage of metabolites and make up the intermembrane space where the proton gradient is created. Finally, in the mitochondrial matrix, we found the mitochondrial DNA, which plays an active role in the stability, replication, and transcription of several mitochondrial genes [28,29]. Mitochondria regulate their shape, size, and number through mitochondrial dynamic processes of fission and fusion commanded by Mitofusins 1 and 2 (Mfn), optic atrophy type-1 (OPA-1; a fusion process protein), and mitochondrial fission-1 (fis-1) and dynamin-related protein 1 (Drp-1) involved in the fission process [30,31].

**Figure 1:**
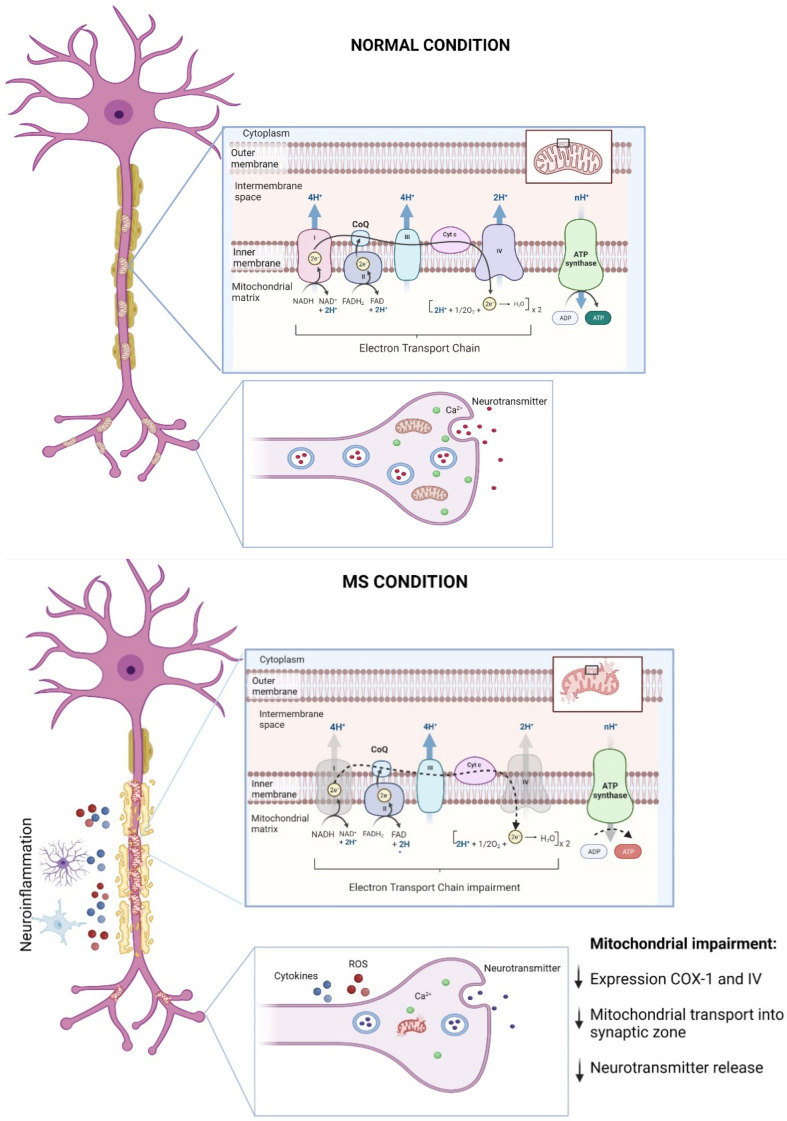
Mitochondrial dysfunction in MS progression. Mitochondria are essential for neuronal function by maintaining bioenergetics through the synthesis of ATP in the electron transport chain (ETC) and by supporting neuron-to-neuron communication by governing [Ca^2+^]_i_ in the presynaptic compartment and then facilitating neurotransmitters release. During MS, the neuroinflammatory environment that characterizes demyelination, i.e., increased ROS levels along with the release of proinflammatory cytokines by glial cells, leads to impaired mitochondrial dynamics, alterations in the synthesis of ATP due to decreases in the expressions of ETC complexes 1 and 4, and decreases in the mitochondria content at presynaptic level, impairing neuronal survival and communication (for details, see the main text). This figure was created in Biorender.

Neurons depend critically on the mitochondrial function to maintain and execute membrane excitability, neurotransmission, and plasticity [32,33,34,35] along with the control of changes in [Ca^2+^]_i_, which contributes to neurotransmitter release [36] (Figure 1). For instance, presynaptic mitochondria can buffer [Ca^2+^]_i_ levels observed in mouse neuromuscular junction (NMJ) [37], which is critical for inducing exocytosis and endocytosis processes [38]. Mitochondrial localization in synaptic regions is also a key factor for proper chemical communication [39]. Studies performed in isolated synaptosomes from a mouse hippocampus exposed to FM1-43 (a lipophilic indicator of synaptic vesicles) showed accumulated synaptic vesicles associated with mitochondria [39]. In this line, Talbot and colleagues [40] showed that stimulus trains triggered on motor terminals induced mitochondrial localization in synaptic zones, where mitochondrial [Ca^2+^] increased and decayed concomitantly with the stimuli [40]. Furthermore, recent reports have confirmed the importance of mitochondrial Ca^2+^ regulation for releasing synaptic vesicles [35,41,42,43]; see seminal articles in [35,43]. Reinforcing this notion, using 3D electron microscopy reconstruction, Smith and colleagues [42] revealed that presynaptic boutons with mitochondria contain more synaptic vesicles compared with boutons lacking mitochondria [42].

As expected, considering its role in synaptic transmission, mitochondrial transport also contributes to neuronal plasticity. Indeed, the proper distribution of mitochondria plays a critical role in dendrites and axonal function [44]. Mitochondria are morphologically more elongated in dendritic zones, where it favors the fusion process; however, during long-term potentiation (LTP), mitochondrial fission predominates in dendritic spines [45]. Significantly, mitochondrial dynamics can influence dendritic arborization and growth [46]. For example, primary hippocampal cultures depolarized by an increase in extracellular K^+^ (KCl 90 mM) showed an increase in dendritic spine number and mitochondrial localization in the same dendritic region after stimulation [46]. Consistently, preventing mitochondrial fission reduced mitochondrial [Ca^2+^], precluding NMDAR-dependent LTP induction in hippocampal slices [45,46]. In fact, pioneer studies proposed Drp1 as a pivotal element orchestrating these plasticity processes [47]. For instance, Purkinje cells with a reduced Drp1 function induced by dominant-negative expression showed abundant elongated mitochondria correlating with a significant decline in the mitochondrial function of dendritic areas affecting dendritic morphology [47]. Furthermore, hippocampal neurons transfected with Drp1-K38A (a dominant-negative form carried a critical mutation in its GTPase domain), presented a decrease in dendritic spines density and mitochondria number [46]. Importantly, both elements were later rescued by Drp1 overexpression [46].

In summary, mitochondrial function and dynamics are essential to proper neuronal performance and communication. Accordingly, mitochondrial dysfunction has been extensively studied in pathological contexts characterized by a loss of neuronal function and survival, being nowadays considered a hallmark of neurodegenerative disorders such as MS.

## 5. Mitochondrial Dysfunction: A Hallmark in the Pathogenesis of MS

As mentioned, MS is an etiologically unknown disease leading to neurological disabilities by demyelination in central white matter observed in young adults between the age of 20–40 [48]. Although studies continue to elucidate the causes, symptoms, and characteristics of MS, recent findings have shown that mitochondrial dysfunction plays a pivotal role in the onset and progression of MS and their related pre-clinical animal models (Figure 1) [49,50,51,52,53,54,55]; for revision see [55].

Mitochondrial impairment has been associated with axonal degeneration followed by demyelination in MS [50,52]. An observation of post mortem MS brain tissues showed an impaired axoplasm, reduced organelle content, and fragmented neurofilaments compared with age-match healthy brain samples [56]. Interestingly, reduced axonal integrity in MS samples was associated with mitochondrial impairment. Here, these findings showed a decreased expression of the electron transport chain (ETC) protein complexes, with complexes I, III, IV, and V being negatively affected. These abnormalities are conducive of impaired mitochondrial respiratory activity [56]. Furthermore, Mahad and colleagues [57] observed a reduction in the expressions of mitochondrial complexes I and IV, which were significantly associated with inflammatory demyelination and microglial activation in MS brain samples [57]. Complementarily, the authors analyzed mitochondrial immunoreactivity using confocal laser microscopy in others cell types from MS brain patients. Here, they observed a massive loss of COX-I expression in the oligodendrocyte and astrocyte populations [57]. Consistently, new reports showed a significant decrease in the activity of mitochondrial complex IV in demyelinated axons from post mortem MS tissue [58]. In addition, it has been reported that mitochondrial content is significantly greater in neurons with demyelinated axons, leading to defective mitochondrial transport [59,60]. Therefore, axonal integrity is essential to mitochondrial transport, and a lack of myelin could negatively prevent mitochondrial transport into synaptic zones, blocking neuronal communication and, finally, triggering MS onset. Interestingly, a recent study provided evidence supporting a putative protective role for this increased mitochondrial content in demyelinated axons [51]. By using an in vitro model of demyelination (i.e., lysolecithin-induced demyelination in cerebellar organotypic cultures), the authors define the accumulation of mitochondria in demyelinated axons as the “axonal response of mitochondria to demyelination” (ARMD; [51]). The authors reported that this ARMD is deficient in axons that finally lose myelin, but when they promote ARMD artificially, the axons were protected from acutely demyelination. These results certainly open new possible roles for the accumulation of mitochondria in axons subjected to demyelination [51].

Oxidative stress has been well documented in many neurodegenerative diseases including MS [16,61]. In this context, oxidative damage induced by an increase in ROS and reactive nitrogen species (NS) has been frequently suggested in MS-based studies in both cerebrospinal fluid samples and animal models [16,61,62,63].

Oxidative stress induces abundant mitochondrial protein nitration associated with mitochondrial bioenergetics failure and mitochondrial DNA (mitoDNA) mutations, and triggering apoptosis in MS brains [50,64,65]. Furthermore, an analysis of cortical slices of MS brain showed a significant presence of oxidative damage, which contributed to axonal demyelination in neurons and oligodendrocytes being more sensitive than astrocytes and microglia [66]. More importantly, the activation of mitochondrial permeability transition pore (mPTP) has been proposed to play a crucial role in oxidative damage and mitochondrial impairment present in MS progression [67]; however, future studies are needed to elucidate mPTP’s role in the pathogenesis of MS.

Although mitochondrial injury remains to be proposed as a hallmark in MS, accumulative studies have consistently demonstrated that mitochondrial dysfunction plays a pivotal role in the MS onset and progression (Figure 1). However, others studies are still necessary to better characterize the impairments in mitochondrial bioenergetics, dynamics, and transport, which have been broadly reported in other neurodegenerative diseases. Closely related, oxidative damage associated with inflammation, mitochondrial injury, and neuronal harm has been extensively reported in MS [16,55,61]. Therefore, examining possible abnormalities in the antioxidant pathways and their contribution to the pathogenesis of MS is a critical aspect. One of the principal antioxidant rescue pathways and its role in MS will be discussed the next section.

## 6. Nrf2 Signaling Network: A Key Player Combatting Oxidative Stress, Mitochondrial Dysfunction, Neuroinflammation, and Neurodegeneration in MS

**Figure 2:**
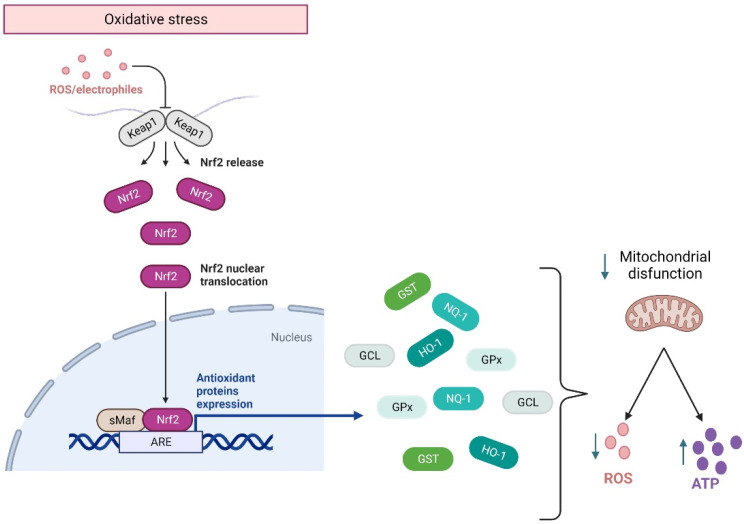
Nrf2 response to oxidative stress. Under an oxidative environment, Keap1 releases Nrf2, which translocates from the cytoplasm to the nucleus, where it binds to the ARE sequence, activating the anti-inflammatory response elements (ARE) pathway. This pathway allows for the transcription of genes that code for the main antioxidant enzymes, which will modulate mitochondrial damage by decreasing ROS and facilitating ATP synthesis. HO-1: heme oxygenase-1. NQO1: H quinone oxidoreductase-1. GST: glutathione S-transferase. GCL: glutamate-cysteine ligase. GPx: glutathione peroxidase. This figure was created in Biorender.

Although in homeostasis, ROS are produced during cellular respiration and neurotransmission, their excessive concentrations are detrimental to cell survival. ROS balance is controlled by the activation of the Nfr2 pathway, which is a transcriptional factor encoded by the gene *NFE2L2* related to the Cap’n’collar family of transcriptional factors. They regulate the basal and stress-inducible expression of over 250 genes containing the antioxidant response elements (ARE) sequence in their promoters [68,69]. These genes constitute a defensive response to ROS overproduction, encoding for heme oxygenase-1 (HO-1), NAD(P) H quinone oxidoreductase-1 (NQO1), glutathione S-transferase (GST), glutamate-cysteine ligase (GCL), and glutathione peroxidase (GPx) [68] (Figure 2). Effectively, in response to ROS overproduction, Nrf2 translocates to the nucleus, activating the Nrf2-ARE pathways. The latter enhances cellular energy and redox potential response, thereby reducing oxidative damage and mitochondrial dysfunction by increasing ATP production and regulating mitochondrial bioenergetics [70] (Figure 2).

During redox imbalance, as observed in MS, the Nrf2 pathway fails to keep ROS at physiological levels [71,72,73,74]. Accordingly, studies indicate that the Nrf2 pathway could be a valuable therapeutic target to ameliorate oxidative stress, mitochondrial impairment, and neuronal damage observed in chronic neurodegenerative diseases [75,76,77]. In this line, post mortem tissue studies from AD patients, show an absence of Nfr2 in the nucleus of hippocampal neurons, while it is normally observed in control patients [75]. Its lack of a nucleus might suggest that Nrf2 is likely not performing its nuclear factor activities, a phenomenon that could be crucial for AD progression [75]. Consistent with this idea, the inhibition of GSK-3β (a kinase involved in tau pathology present in AD), along with lithium administration, increases the transcriptional activity of Nrf2 [78]. The latter combined with the observation that Nrf2^−/−^ mice exhibit higher levels of hippocampal oxidative damage and inflammation than wild-type mice [79] implicates that Nrf2 signaling impairment is critical for neuronal damage observed in AD.

On the other hand, mounting evidence has suggested that the Nrf2 factor could contribute to chronic neuroinflammation [80,81]. Indeed, NFκB-dependent responses, another major pathway activated by oxidative stress, are regulated by Nrf2 [82,83], whereas its activation represents a critical antioxidant checkpoint for astrocytes, conferring them neuroprotective properties during neuroinflammation [84]. Consistent with this, Nrf2-dependent genes are activated in stressed astrocytes [85], whereas Nrf2^−/−^ microglia fail to promote the expressions of HO-1 and NQO1 [86]. More relevant, Nrf2^−/−^ microglia shift their reactive profile towards increased production of IL-6, IL-1β, and iNOS and reduced phagocytic capacity [86]. This evidence along with other similar studies permits us to propose Nrf2 as a potential pharmacological target for ameliorating neurodegenerative changes induced by neuroinflammation [87].

Evidence from animal studies has reinforced the possible dialogue of the Nfr2 pathway with inflammation and redox homeostasis. Indeed, the treatment with dimethyl fumarate (DMF) prevents inflammation and oxidative damage in different models of neurodegenerative diseases, such as AD [88] and MS [89,90]. The alleviative influence of DMF on cytokine and ROS production and the migratory activity of immune cells at the blood–brain barrier (BBB) have been linked to the activation of the Nrf2 pathway. Since 2013, DMF has been approved by the U.S. Food and Drug Administration (FDA) as a treatment option for adults with RRMS, and part of its beneficial effects on MS have been hypothesized to occur via Nrf2 activation [91,92,93]. The latter has been inferred from studies in two commonly used animal models of MS: the experimental autoimmune encephalomyelitis (EAE) and treatment with cuprizone [94]. Nrf2 and its downstream target proteins increase their levels after 1–3 weeks but decrease after five weeks of cuprizone treatment, indicating that Nrf2 expression mimics the disease progression [95]. In the same line, Nrf2^−/−^ mice treated with cuprizone show increased apoptosis of oligodendrocytes, neuroinflammation, and axonal damage compared with wild-type controls [96]. In addition, Nrf2^−/−^ animals display a higher susceptibility towards cuprizone within the anterior white commissure, a relatively insensitive structure to this drug in wild-type animals [96]. More importantly, DMF treatment reduces symptom severity in EAE mice and preserves myelin content and axon density, a response being lost in Nrf2^−/−^ mice [97]. Although recent evidence indicates that DMF could reduce the activity of pro-inflammatory microglia [98], the molecular and cellular mechanisms underlying these phenomena and how glial cells participate in them in MS remain unknown.

## 7. Large-Pore Channels: A Possible Link between Glial Cell Dysfunction and Nrf2 in Multiple Sclerosis

Hemichannels and pannexons belong to the large-pore channel family, and mounting evidence suggests that their activation leads to glial cell dysfunction in diverse neuroinflammatory conditions [99]. In contrast with most plasma membrane channels that selectively permeate ions such as K^+^, Na^+^, and Cl^−^, large-pore channels constitute conduits for the passage not only of ions but also small molecules, as they have greater pore diameters than selective ion channels. Hemichannels are composed of six connexin monomers around a central pore that allow for the passage of ions and small molecules between the cytosol and the extracellular space [100]. On the side, pannexons result from the oligomerization of seven pannexins, a three-member family of proteins (Panx1-3) with equivalent secondary and tertiary structures to connexins with the ability to form plasma membrane channels [101]. In the CNS, astrocytes and microglia express functional connexin 43 (Cx43) hemichannels and Panx1 channels, and other studies also have observed microglial connexin 32 (Cx32) hemichannels [102,103]. Although neurons and oligodendrocytes express Panx1 channels, the existence of hemichannels in these cells has not been consistently determined [104,105].

In the brain, glial cell hemichannels and pannexons permit the release of gliotransmitters that have been found crucial for synaptic transmission and plasticity as well as behavior and memory [106,107,108,109,110]. Nonetheless, during pathological conditions, the exacerbated activity of these channels in microglia, astrocytes, and oligodendrocytes has been linked to the homeostatic disturbances occurring in the pathogenesis and progression of different brain diseases [99,111,112]. This idea comes from studies demonstrating that osmotic and ionic imbalances induced by the uncontrolled influx of Na^2+^ and Cl^−^ through hemichannels/pannexons could result in further aquaporin-mediated cell swelling and plasma membrane breakdown [113,114]. In addition, it has been proposed that because hemichannels/pannexons are permeable to Ca^2+^, their uncontrolled opening could lead to Ca^2+^ overload and the consequent production of free radicals, lipid peroxidation, and plasma membrane damage [99]. Alternatively, exacerbated hemichannel/pannexon activity could also induce the release of potentially harmful molecules for neighboring cells, such as glutamate, ATP, and D-serine [99].

**Figure 3:**
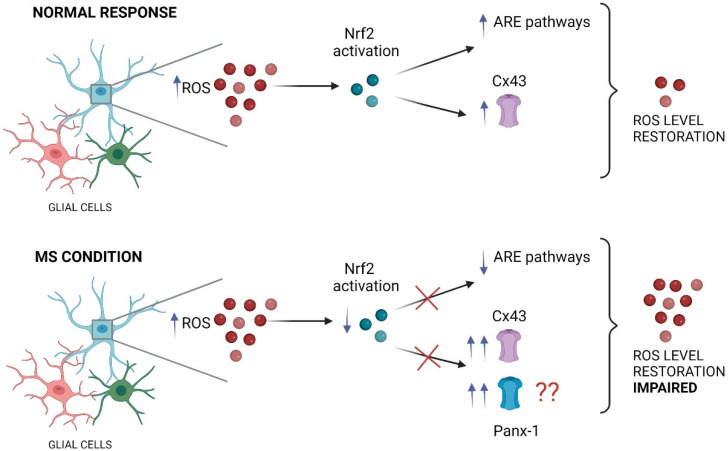
The impact of impaired Nrf2 pathway on glial large pore channels during MS. Under neuroinflammatory conditions, the increase in ROS triggers typically the activation of the Nrf2 transcription factor in glial cells (such as astrocytes and microglia), inducing the anti-inflammatory response element (ARE) pathway activation that finally restore the ROS levels. In these conditions, Nrf2 maintains an inhibitory tone on Cx43 hemichannels, keeping their activity in a physiological range. In contrast, during MS the proper function of the Nrf2 transcription factor is impaired, which result in decreased activation of ARE pathways accompanied of persistent and exacerbated opening of large pore channels, including Cx43 hemichannels and likely Panx1 channels in glial cells (see the main text). This figure was created in Biorender.

Recent studies have shed light on a potential molecular mechanism linking MS, Nrf2 signaling, and glial connexins. Using the EAE mice, Shijie and colleagues [115] demonstrated that treatment with carbenoxolone (CBX), a general blocker of gap junction channels, hemichannels, and pannexons, attenuated EAE clinical symptoms [115]. The ameliorative effects of CBX were proposed to occur by reducing the release of glutamate from activated microglia through these large-pore channels. Interestingly, it has been shown that the activation of the Nrf2/HO-1/CO pathway inhibits the expression of Cx43 in spinal astrocytes, reducing neuropathic pain [116]. This evidence supports a putative causal relationship between the activation of the Nrf2 pathway and the modification of Cx43 expression, the most ubiquitous connexin-forming hemichannels in astrocytes [116]. In the same line, Cx43 triggers protective effects in diabetes via activation of the Nrf2/ARE pathway [117,118]. Reinforcing the notion of Nrf2 activation/Cx43 regulation, protective effects of bone marrow mesenchymal stem cells against intracerebral hemorrhage have been linked to Cx43 upregulation along with Nrf2 nuclear translocation in astrocytes [119]. Notably, in this study, the knockdown of Cx43 by siRNA restrained Nrf2 nuclear translocation likely because Cx43 and Nrf2 seem to establish direct protein interactions. Altogether this evidence suggests a reciprocal regulation of Nrf2 signaling and Cx43 with significant potential consequences for glial cell function (Figure 3).

Despite information directly connecting the opening of hemichannels with the pathogenesis and progression of MS lacking, a couple of studies have proposed a role for pannexons. Pioneering experiments by Negoro and colleagues [120] found that EAE mice exhibit increased expressions of Panx1 and Cx43 in the bladder mucosa linked to dysfunctional micturition, a common disorder in MS [120]. Notably, the ablation of Panx1 reduced bladder dysfunction and prevented Cx43 and IL-1β upregulation in EAE mice. Consistent with this evidence, multiple studies by Meier’s Laboratory have shown that probenecid, a Panx1 channel blocker, counteracts clinical symptoms and inflammation observed in several MS models [121,122,123]. However, the molecular and cellular mechanisms behind this protective action of probenecid are unknown [124].

## 8. Concluding Remarks

A pending aspect in the multiple sclerosis research field is to unravel the relationship between the glial neuroinflammation, demyelination, and the failed activation of endogenous responsive mechanisms such as the Nrf2-signaling, which under normal conditions play critical roles in preventing mitochondrial failure, oxidative stress, and neuroinflammation. Current evidence suggests a regulation of Nrf2 signaling on the activity of Cx43-based channels and vice versa. In the same line, some studies permit us to speculate that another large-pore channel, those based on the assembly of Panx-1, could also contribute to the Nrf2 activation-dependent response. Although there is some agreement that neuroinflammation, oxidative stress, mitochondrial dysfunction, and Nrf2-impaired signaling are vital elements in the development of neurodegenerative diseases such as multiple sclerosis, there is still no certainty on the cellular/molecular targets involved. Here, we propose that the change in permeability of glial connexin and pannexin-based channels represents a critical aspect of this problem. Future studies must elucidate whether the opening of hemichannels and pannexons in the CNS could contribute to inflammation and the redox imbalance observed in MS by a mechanism involving the dysfunction of Nrf2 signaling.
